# Supplementary material for: A UMLS-based spell checker for natural language processing in vaccine safety
Source: BMC Med Inform Decis Mak. 2007 Feb 12;7:3. doi: 10.1186/1472-6947-7-3 (PMC1805499; doi:10.1186/1472-6947-7-3)
Supplement: Additional file 13 — RAP application directory. Sets up directory for RAP (RDF application for PHP) source code [file 1472-6947-7-3-S13.gz › rap/doc/phpdoc/__filesource/fsource_model__modelDbStore.php.html]

xml version="1.0" encoding="iso-8859-1"?


File Source for DbStore.php


# Source for file DbStore.php

Documentation is available at DbStore.php

```
1. <?php

3. // ----------------------------------------------------------------------------------
4. // Class: DbStore
5. // ----------------------------------------------------------------------------------


9. /**
10. * DbStore is a persistent store of RDF data using relational database technology.
11. * DbStore uses ADOdb Library for PHP V3.60 (http://php.weblogs.com/ADODB),
12. * which allows to connect to multiple databases in a portable manner.
13. * This class also provides methods for creating tables for MsAccess, MySQL, and MS SQL Server.
14. * If you want to use other databases, you will have to create tables by yourself
15. * according to the abstract database schema described in the API documentation.
16. *
17. * <BR><BR>History:<UL>
18. * <LI>05-30-2005                 : added dataset support
19. * <LI>12-06-2004                : improved namespace handling added (tobias.gauss@web.de)</LI>
20. * <LI>10-19-2004                : _isSetup_MSSQL() added. tobias.gauss@web.de</LI>
21. * <LI>09-02-2004                 : _isSetup_MySql() and _isSetupMsAccess() added. ggrimnes@csd.abdn.ac.uk/ tobias.gauss@web.de</LI>
22. * <LI>06-17-2003                : First version of this class</LI>
23. *
24. *
25. * @version  V0.9.1
26. * @author   Radoslaw Oldakowski <radol@gmx.de>
27. * @author   Daniel Westphal (http://www.d-westphal.de)
28. *
29. * @package model
30. * @access    public
31. */


34. class DbStore extends Object{

36. /**
37. * Database connection object
38. *
39. * @var     object ADOConnection
40. * @access    private
41. */
42. var $dbConn;


45. /**
46. * Constructor:
47. * Set the database connection with the given parameters.
48. *
49. * @param   string   $dbDriver
50. * @param   string   $host
51. * @param   string   $dbName
52. * @param   string   $user
53. * @param   string   $password
54. * @access    public
55. */
56. function DbStore ($dbDriver=ADODB_DB_DRIVER, $host=ADODB_DB_HOST, $dbName=ADODB_DB_NAME,
57. $user=ADODB_DB_USER, $password=ADODB_DB_PASSWORD) {

59. // include DBase Package
60. require_once(RDFAPI_INCLUDE_DIR.PACKAGE_DBASE);

62. // create a new connection object
63. $this->dbConn =& ADONewConnection($dbDriver);

65. // connect to database
66. $this->dbConn->connect($host, $user, $password, $dbName);

68. // optimized for speed
69. $this->dbConn->setFetchMode(ADODB_FETCH_NUM);
70. $ADODB_COUNTRECS = FALSE;

72. //activate the ADOdb DEBUG mode
73. if (ADODB_DEBUG_MODE =='1')
74. $this->dbConn->debug = TRUE;
75. }


78. /**
79. * Create tables and indexes for the given database type.
80. * Currently supported: MsAccess and MySQL.
81. * If you want to use other databases, you will have to create tables by yourself
82. * according to the abstract <a href="database_schema.html">database schema</a>
83. * described in the API documentation.
84. *
85. * @param   string  $databaseType
86. * @throws    PhpError
87. * @access    public
88. */
89. function createTables($databaseType) {

91. if (!strcasecmp($databaseType, 'MsAccess'))
92. $this->_createTables_MsAccess();
93. elseif (!strcasecmp($databaseType, 'MySQL'))
94. $this->_createTables_MySql();
95. elseif (!strcasecmp($databaseType, 'MSSQL'))
96. $this->_createTables_mssql();
97. else {
98. $errmsg = RDFAPI_ERROR . "(class: DbStore; method: createTables('$databaseType')):
99. Currently only MsAcces, MySQL and MSSQL supported.";
100. trigger_error($errmsg, E_USER_ERROR);
101. }
102. }


105. /**
106. * List all DbModels stored in the database.
107. *
108. * @return  array
109. * @throws    SqlError
110. * @access    public
111. */
112. function listModels() {

114. $recordSet =& $this->dbConn->execute("SELECT modelURI, baseURI
115. FROM models");
116. if (!$recordSet)
117. echo $this->dbConn->errorMsg();
118. else {
119. $models = array();
120. $i=0;
121. while (!$recordSet->EOF) {

123. $models[$i]['modelURI'] = $recordSet->fields[0];
124. $models[$i]['baseURI'] = $recordSet->fields[1];

126. ++$i;
127. $recordSet->moveNext();
128. }
129. return $models;
130. }
131. }


134. /**
135. * Check if the DbModel with the given modelURI is already stored in the database
136. *
137. * @param   string   $modelURI
138. * @return  boolean
139. * @throws    SqlError
140. * @access    public
141. */
142. function modelExists($modelURI) {

144. $res =& $this->dbConn->execute("SELECT COUNT(*) FROM models
145. WHERE modelURI = '" .$modelURI ."'");
146. if (!$res)
147. echo $this->dbConn->errorMsg();
148. else {
149. if (!$res->fields[0])
150. return FALSE;
151. return TRUE;
152. }
153. }


156. /**
157. * Create a new instance of DbModel with the given $modelURI and
158. * load the corresponding values of modelID and baseURI from the database.
159. * Return FALSE if the DbModel does not exist.
160. *
161. * @param   string   $modelURI
162. * @return  object DbModel
163. * @access    public
164. */
165. function getModel($modelURI) {

167. if (!$this->modelExists($modelURI))
168. return FALSE;
169. else {
170. $modelVars =& $this->dbConn->execute("SELECT modelURI, modelID, baseURI
171. FROM models
172. WHERE modelURI='" .$modelURI ."'");

174. return new DbModel($this->dbConn, $modelVars->fields[0],
175. $modelVars->fields[1], $modelVars->fields[2]);
176. }
177. }


180. /**
181. * Create a new instance of DbModel with the given $modelURI
182. * and insert the DbModel variables into the database.
183. * Return FALSE if there is already a model with the given URI.
184. *
185. * @param   string   $modelURI
186. * @param   string   $baseURI
187. * @return  object DbModel
188. * @throws  SqlError
189. * @access    public
190. */
191. function getNewModel($modelURI, $baseURI=NULL) {

193. if ($this->modelExists($modelURI))
194. return FALSE;
195. else {
196. $modelID = $this->_createUniqueModelID();

198. $rs =& $this->dbConn->execute("INSERT INTO models
199. VALUES ('" .$modelID ."',
200. '" .$modelURI ."',
201. '" .$baseURI ."')");
202. if (!$rs)
203. $this->dbConn->errorMsg();
204. else
205. return new DbModel($this->dbConn, $modelURI, $modelID, $baseURI);
206. }
207. }


210. /**
211. * Store a MemModel or another DbModel from a different DbStore in the database.
212. * Return FALSE if there is already a model with modelURI matching the modelURI
213. * of the given model.
214. *
215. * @param   object Model  &$model
216. * @param   string $modelURI
217. * @return  boolean
218. * @access    public
219. */
220. function putModel(&$model, $modelURI=NULL) {

222. if (!$modelURI) {
223. if (is_a($model, 'MemModel'))
224. $modelURI = 'DbModel-' .$this->_createUniqueModelID();
225. else
226. $modelURI = $model->modelURI;
227. }else
228. if ($this->modelExists($modelURI))
229. return FALSE;


232. $newDbModel = $this->getNewModel($modelURI, $model->getBaseURI());
233. $newDbModel->addModel($model);
234. }


237. /**
238. * Close the DbStore.
239. * !!! Warning: If you close the DbStore all active instances of DbModel from this
240. * !!!          DbStore will lose their database connection !!!
241. *
242. * @access    public
243. */
244. function close() {

246. $this->dbConn->close();
247. unset($this);
248. }


251. // =============================================================================
252. // **************************** private methods ********************************
253. // =============================================================================


259. /**
260. * Create a unique ID for the DbModel to be insert into the models table.
261. * This method was implemented because some databases do not support auto-increment.
262. *
263. * @return  integer
264. * @access    private
265. */
266. function _createUniqueModelID() {

268. $maxModelID =& $this->dbConn->GetOne('SELECT MAX(modelID) FROM models');
269. return ++$maxModelID;
270. }

272. /**
273. * Create a unique ID for the dataset to be insert into the datasets table.
274. * This method was implemented because some databases do not support auto-increment.
275. *
276. * @return  integer
277. * @access    private
278. */
279. function _createUniqueDatasetID() {

281. $maxDatasetID =& $this->dbConn->GetOne('SELECT MAX(datasetId) FROM datasets');
282. return ++$maxDatasetID;
283. }


286. /**
287. * Create tables and indexes for MsAccess database
288. *
289. * @throws  SqlError
290. * @access    private
291. */
292. function _createTables_MsAccess() {

294. $this->dbConn->startTrans();

296. $this->dbConn->execute('CREATE TABLE models
297. (modelID long primary key,
298. modelURI varchar not null,
299. baseURI varchar)');

301. $this->dbConn->execute('CREATE UNIQUE INDEX m_modURI_idx ON models (modelURI)');

303. $this->dbConn->execute('CREATE TABLE statements
304. (modelID long,
305. subject varchar,
306. predicate varchar,
307. object Memo,
308. l_language varchar,
309. l_datatype varchar,
310. subject_is varchar(1),
311. object_is varchar(1),
312. primary key (modelID, subject, predicate, object,
313. l_language, l_datatype))');

315. $this->dbConn->execute('CREATE INDEX s_mod_idx ON statements (modelID)');
316. $this->dbConn->execute('CREATE INDEX s_sub_idx ON statements (subject)');
317. $this->dbConn->execute('CREATE INDEX s_pred_idx ON statements (predicate)');
318. $this->dbConn->execute('CREATE INDEX s_obj_idx ON statements (object)');

320. $this->dbConn->execute('CREATE TABLE namespaces
321. (modelID long,
322. namespace varchar,
323. prefix varchar,
324. primary key (modelID, namespace, prefix))');

326. $this->dbConn->execute('CREATE INDEX n_name_idx ON namespaces (namespace)');
327. $this->dbConn->execute('CREATE INDEX n_pref_idx ON namespaces (prefix)');

329. $this->dbConn->execute("CREATE TABLE datasets
330. (datasetName varchar,
331. defaultModelUri varchar,
332. primary key (datasetName))");

334. $this->dbConn->execute('CREATE INDEX nGS_idx1 ON datasets (datasetName)');


337. $this->dbConn->execute("CREATE TABLE `dataset_model` (
338. datasetName varchar,
339. modelId long,
340. graphURI varchar,
341. PRIMARY KEY  (modelId,datasetName))");


344. if (!$this->dbConn->completeTrans())
345. echo $this->dbConn->errorMsg();
346. }


349. /**
350. * Create tables and indexes for MySQL database
351. *
352. * @throws  SqlError
353. * @access    private
354. */
355. function _createTables_MySql() {

357. $this->dbConn->startTrans();

359. $this->dbConn->execute("CREATE TABLE models
360. (modelID bigint NOT NULL,
361. modelURI varchar(255) NOT NULL,
362. baseURI varchar(255) DEFAULT '',
363. primary key (modelID))");

365. $this->dbConn->execute('CREATE UNIQUE INDEX m_modURI_idx ON models (modelURI)');

367. $this->dbConn->execute("CREATE TABLE statements
368. (modelID bigint NOT NULL,
369. subject varchar(255) NOT NULL,
370. predicate varchar(255) NOT NULL,
371. object text,
372. l_language varchar(255) DEFAULT '',
373. l_datatype varchar(255) DEFAULT '',
374. subject_is varchar(1) NOT NULL,
375. object_is varchar(1) NOT NULL)");

377. $this->dbConn->execute("CREATE TABLE namespaces
378. (modelID bigint NOT NULL,
379. namespace varchar(255) NOT NULL,
380. prefix varchar(255) NOT NULL,
381. primary key (modelID,namespace))");

383. $this->dbConn->execute("CREATE TABLE `dataset_model` (
384. `datasetName` varchar(255) NOT NULL default '0',
385. `modelId` bigint(20) NOT NULL default '0',
386. `graphURI` varchar(255) NOT NULL default '',
387. PRIMARY KEY  (`modelId`,`datasetName`))");

389. $this->dbConn->execute("CREATE TABLE `datasets` (
390. `datasetName` varchar(255) NOT NULL default '',
391. `defaultModelUri` varchar(255) NOT NULL default '0',
392. PRIMARY KEY  (`datasetName`),
393. KEY `datasetName` (`datasetName`))");

395. $this->dbConn->execute('CREATE INDEX s_mod_idx ON statements (modelID)');
396. $this->dbConn->execute('CREATE INDEX n_mod_idx ON namespaces (modelID)');

398. $this->dbConn->execute('CREATE INDEX s_sub_pred_idx ON statements
399. (subject(200),predicate(200))');
400. $this->dbConn->execute('CREATE INDEX s_obj_idx ON statements (object(250))');

402. if (!$this->dbConn->completeTrans())
403. echo $this->dbConn->errorMsg();
404. }

406. /**
407. * Create tables and indexes for MSSQL database
408. *
409. * @throws  SqlError
410. * @access    private
411. */
412. function _createTables_mssql(){

414. $this->dbConn->startTrans();

416. $this->dbConn->execute("CREATE TABLE [dbo].[models] (
417. [modelID] [int] NOT NULL ,
418. [modelURI] [nvarchar] (200) COLLATE SQL_Latin1_General_CP1_CI_AS NULL ,
419. [baseURI] [nvarchar] (200) COLLATE SQL_Latin1_General_CP1_CI_AS NULL
420. ) ON [PRIMARY]");

422. $this->dbConn->execute("CREATE TABLE [dbo].[statements] (
423. [modelID] [int] NOT NULL ,
424. [subject] [nvarchar] (200) COLLATE SQL_Latin1_General_CP1_CI_AS NULL ,
425. [predicate] [nvarchar] (200) COLLATE SQL_Latin1_General_CP1_CI_AS NULL ,
426. [object] [text] COLLATE SQL_Latin1_General_CP1_CI_AS NULL ,
427. [l_language] [nvarchar] (50) COLLATE SQL_Latin1_General_CP1_CI_AS NULL ,
428. [l_datatype] [nvarchar] (50) COLLATE SQL_Latin1_General_CP1_CI_AS NULL ,
429. [subject_is] [nchar] (1) COLLATE SQL_Latin1_General_CP1_CI_AS NULL ,
430. [object_is] [nchar] (1) COLLATE SQL_Latin1_General_CP1_CI_AS NULL
431. ) ON [PRIMARY] TEXTIMAGE_ON [PRIMARY]");


434. $this->dbConn->execute("CREATE TABLE [dbo].[namespaces] (
435. [modelID] [int] NOT NULL ,
436. [namespace] [nvarchar] (200) COLLATE SQL_Latin1_General_CP1_CI_AS NOT NULL ,
437. [prefix] [nvarchar] (200) COLLATE SQL_Latin1_General_CP1_CI_AS NULL ,
438. ) ON [PRIMARY]");

440. $this->dbConn->execute("ALTER TABLE [dbo].[models] WITH NOCHECK ADD
441. CONSTRAINT [PK_models] PRIMARY KEY  CLUSTERED
442. (
443. [modelID]
444. )  ON [PRIMARY] ");
445. $this->dbConn->execute("ALTER TABLE [dbo].[namespaces] WITH NOCHECK ADD
446. CONSTRAINT [PK_namespaces] PRIMARY KEY  CLUSTERED
447. (
448. [modelID],[namespace]
449. )  ON [PRIMARY] ");

451. $this->dbConn->execute("CREATE  INDEX [joint index on subject and predicate] ON [dbo].[statements]([subject], [predicate]) ON [PRIMARY]");


454. if (!$this->dbConn->completeTrans())
455. echo $this->dbConn->errorMsg();

457. }


460. /**
461. * Checks if tables are setup for RAP
462. *
463. * @param   string  $databaseType
464. * @throws SqlError
465. * @access public
466. ***/
467. function isSetup($databaseType="MySQL") {
468. if ($databaseType=="MySQL")
469. return $this->_isSetup_MySql();
470. if ($databaseType=="MSSQL")
471. return $this->_isSetup_MSSQL();
472. else {
473. if ($databaseType=='MsAccess'){
474. return $this->_isSetup_MsAccess();
475. }else{
476. $errmsg=RDFAPI_ERROR."(class: DbStore; method isSetup('$databaseType')):\nCurrently only MySQL, MsAccess and MSSQL are supported!";
477. trigger_error($errmsg, E_USER_ERROR);}
478. }
479. }

481. /**
482. * Checks if tables are setup for RAP (MySql)
483. *
484. * @throws SqlError
485. * @access private
486. ***/
487. function _isSetup_MySql() {
488. $recordSet =& $this->dbConn->execute("SHOW TABLES");
489. if (!$recordSet)
490. echo $this->dbConn->errorMsg();
491. else {
492. $tables = array();
493. while (!$recordSet->EOF) {

495. $tables[]= $recordSet->fields[0];

497. if(isset($i)){++$i;}
498. $recordSet->moveNext();
499. }
500. if (in_array("models",$tables) && in_array("statements",$tables)&& in_array("namespaces",$tables)) return true;
501. }
502. return false;

504. }


507. /**
508. * Checks if tables are setup for RAP (MsAccess)
509. *
510. * @throws SqlError
511. * @access private
512. ***/
513. function _isSetup_MsAccess() {
514. $tables =& $this->dbConn->MetaTables();
515. if (!$tables)
516. echo $this->dbConn->errorMsg();
517. if (count($tables)==0){
518. return false;}
519. else {
520. if (in_array("models",$tables) && in_array("statements",$tables) && in_array("namespaces",$tables)){ return true;
521. }else{return false;}
522. }
523. }

525. /**
526. * Checks if tables are setup for RAP (MSSQL)
527. *
528. * @throws SqlError
529. * @access private
530. ***/
531. function _isSetup_MSSQL() {
532. $tables =& $this->dbConn->MetaTables();
533. if (!$tables)
534. echo $this->dbConn->errorMsg();
535. if (count($tables)==0){
536. return false;}
537. else {
538. if (in_array("models",$tables) && in_array("statements",$tables) && in_array("namespaces",$tables)){ return true;
539. }else{return false;}
540. }
541. }


544. /**
545. * Create a new instance of DatasetDb with the given $datasetName
546. * and insert the DatasetDb variables into the database.
547. * Return FALSE if there is already a model with the given URI.
548. *
549. * @param   $datasetName string
550. * @return  object DatasetDB
551. * @throws  SqlError
552. * @access    public
553. */
554. function & getNewDatasetDb($datasetName)
555. {

557. require_once(RDFAPI_INCLUDE_DIR . PACKAGE_DATASET);

559. if ($this->datasetExists($datasetName))
560. return FALSE;
561. else
562. {
563. $defaultModelUri=uniqid('http://rdfapi-php/dataset_defaultmodel_');
564. $defaultModel=$this->getNewModel($defaultModelUri);

566. $rs =& $this->dbConn->execute("INSERT INTO datasets
567. VALUES ('" .$datasetName ."',
568. '" .$defaultModelUri."')");

570. if (!$rs)
571. $this->dbConn->errorMsg();
572. else
573. $return=new DatasetDb(&$this->dbConn,&$this,$datasetName);
574. return ($return);
575. }
576. }

578. /**
579. * Check if the Dataset with the given $datasetName is already stored in the database
580. *
581. * @param   $datasetName string
582. * @return  boolean
583. * @throws    SqlError
584. * @access    public
585. */
586. function datasetExists($datasetName) {

588. $res =& $this->dbConn->execute("SELECT COUNT(*) FROM datasets
589. WHERE datasetName = '" .$datasetName ."'");
590. if (!$res)
591. echo $this->dbConn->errorMsg();
592. else {
593. if (!$res->fields[0])
594. return FALSE;
595. return TRUE;
596. }
597. }


600. /**
601. * Create a new instance of DatasetDb with the given $datasetName and
602. * load the corresponding values from the database.
603. * Return FALSE if the DbModel does not exist.
604. *
605. * @param   $datasetId string
606. * @return  object DatasetDb
607. * @access    public
608. */
609. function getDatasetDb($datasetName) {

611. require_once(RDFAPI_INCLUDE_DIR . PACKAGE_DATASET);

613. if (!$this->datasetExists($datasetName))
614. return FALSE;
615. else
616. {
617. $return=new DatasetDb(&$this->dbConn,&$this,$datasetName);
618. return ($return);
619. }
620. }

622. /**
623. * Create a new instance of namedGraphDb with the given $modelURI and graphName and
624. * load the corresponding values of modelID and baseURI from the database.
625. * Return FALSE if the DbModel does not exist.
626. *
627. * @param   $modelURI string
628. * @param   $graphName string
629. * @return  object NamedGraphMem
630. * @access    public
631. */
632. function getNamedGraphDb($modelURI, $graphName)
633. {
634. require_once(RDFAPI_INCLUDE_DIR . PACKAGE_DATASET);

636. if (!$this->modelExists($modelURI))
637. return FALSE;
638. else {
639. $modelVars =& $this->dbConn->execute("SELECT modelURI, modelID, baseURI
640. FROM models
641. WHERE modelURI='" .$modelURI ."'");

643. return new NamedGraphDb($this->dbConn, $modelVars->fields[0],
644. $modelVars->fields[1], $graphName ,$modelVars->fields[2]);
645. }
646. }

648. /**
649. * Create a new instance of namedGraphDb with the given $modelURI and graphName
650. * and insert the DbModel variables into the database (not the graphName. This
651. * is only stored persistently, when added to dataset).
652. * Return FALSE if there is already a model with the given URI.
653. *
654. * @param   $modelURI string
655. * @param      $graphName string
656. * @param   $baseURI string
657. * @return  object namedGraphDb
658. * @throws  SqlError
659. * @access    public
660. */
661. function getNewNamedGraphDb($modelURI, $graphName, $baseURI=NULL) {

663. if ($this->modelExists($modelURI))
664. return FALSE;
665. else {
666. $modelID = $this->_createUniqueModelID();

668. $rs =& $this->dbConn->execute("INSERT INTO models
669. VALUES ('" .$modelID ."',
670. '" .$modelURI ."',
671. '" .$baseURI ."')");
672. if (!$rs)
673. $this->dbConn->errorMsg();
674. else
675. return new NamedGraphDb($this->dbConn, $modelURI, $modelID, $graphName, $baseURI);
676. }
677. }

679. /**
680. * Removes the graph with all statements from the database.
681. * Warning: A single namedGraph can be added to several datasets. So it'll be
682. * removed from all datasets.
683. *
684. * @param   $modelURI string
685. * @return  boolean
686. * @throws  SqlError
687. * @access    public
688. */
689. function removeNamedGraphDb($modelURI)
690. {
691. if (!$this->modelExists($modelURI))
692. return FALSE;

694. $modelID = $this->dbConn->GetOne("SELECT modelID FROM models WHERE modelURI='".$modelURI."'");

696. $this->dbConn->execute("DELETE FROM models WHERE modelID=".$modelID);
697. $this->dbConn->execute("DELETE FROM dataset_model WHERE modelId=".$modelID);
698. $this->dbConn->execute("DELETE FROM statements WHERE modelID=".$modelID);

700. return true;
701. }

703. } // end: Class DbStore
704. ?>
```

Documentation generated on Thu, 7 Jul 2005 13:41:25 +0200 by phpDocumentor 1.3.0RC3
